# Advancing insights into microgravity induced muscle changes using *Caenorhabditis elegans* as a model organism

**DOI:** 10.1038/s41526-024-00418-z

**Published:** 2024-07-26

**Authors:** Laura J. Beckett, Philip M. Williams, Li Shean Toh, Volker Hessel, Lukas Gerstweiler, Ian Fisk, Luis Toronjo-Urquiza, Veeren M. Chauhan

**Affiliations:** 1 School of Pharmacy, University of Nottingham, Nottingham, UK; 2 School of Chemical Engineering, North Terrace Campus, The University of Adelaide, Adelaide, SA Australia; 3 International Flavour Research Centre, Division of Food, Nutrition and Dietetics, University of Nottingham, Sutton Bonington Campus, Loughborough, UK; 4 International Flavour Research Centre (Adelaide), School of Agriculture, Food and Wine and Waite Research Institute, The University of Adelaide, Adelaide, SA Australia

**Keywords:** Developmental biology, Physiology, Chemical biology

## Abstract

Spaceflight presents significant challenges to the physiological state of living organisms. This can be due to the microgravity environment experienced during long-term space missions, resulting in alterations in muscle structure and function, such as atrophy. However, a comprehensive understanding of the adaptive mechanisms of biological systems is required to devise potential solutions and therapeutic approaches for adapting to spaceflight conditions. This review examines the current understanding of the challenges posed by spaceflight on physiological changes, alterations in metabolism, dysregulation of pathways and the suitability and advantages of using the model organism *Caenorhabditis elegans* nematodes to study the effects of spaceflight. Research has shown that changes in the gene and protein composition of nematodes significantly occur across various larval stages and rearing environments, including both microgravity and Earth gravity settings, often mirroring changes observed in astronauts. Additionally, the review explores significant insights into the fundamental metabolic changes associated with muscle atrophy and growth, which could lead to the development of diagnostic biomarkers and innovative techniques to prevent and counteract muscle atrophy. These insights not only advance our understanding of microgravity-induced muscle atrophy but also lay the groundwork for the development of targeted interventions to mitigate its effects in the future.

## Introduction: understanding microgravity-induced muscle atrophy; mechanisms, biomarkers and implications for astronaut health

### Microgravity and its effects

Microgravity refers to when the apparent gravity experienced by an object, or a person is significantly reduced^1^. This condition is commonly experienced in space environments, during orbital missions around Earth. Prolonged exposure to microgravity leads to the weakening of muscles and bones^2^. Astronauts in spaceflight can experience muscle atrophy (muscle loss) and bone density loss, especially in weight-bearing areas like the legs and spine. In microgravity, the heart does not have to work as hard to pump blood against gravity. Astronauts often experience cardiovascular deconditioning, including a decrease in heart muscle mass, reduced heart rate and altered blood pressure regulation^3^. Over time, this can result in reduced aerobic ability. Previous research conducted by NASA (Scott Smith and colleagues^4^) demonstrated that it is possible to mitigate muscle loss in space with countermeasures, such as exercise programmes, specialised diets and other interventions. However, without such countermeasures astronauts could lose up to 50% of muscle mass on long-duration missions^5^. It is important to note that countermeasures like these do not necessarily solve the issue at hand, with astronauts still losing between 10% and 20% muscle mass up until their return to Earth and under 1 *g* conditions^6^. Recognising the effects of microgravity is becoming increasingly important and the use of nematodes as model organisms proves invaluable in elucidating the physiological responses to altered gravitational conditions, supplying a unique perspective that significantly contributes to broader explanation of space-related challenges.

### An introduction to muscle atrophy

Atrophy, a condition marked by cellular regression and a reduction in structural volume, is characterised by the loss of myofibrillar proteins. Consequently, muscle fibres undergo a shrinkage and alterations in composition. Human muscle atrophy stems from reduced protein synthesis, increased protein degradation and changes in cellular signalling. This results in muscle wasting as the ubiquitin-proteasome system (UPS) intensifies protein degradation processes, causing muscle fibres to lose proteins^7^. Specifically, muscle-specific E3 ubiquitin ligases like MuRF1 and MAFbx become upregulated during atrophy on Earth and in spaceflight, targeting structural proteins and contractile elements within muscle fibres^8^.

Atrophy activation involves several mechanisms, including the ATP-dependent ubiquitin-proteasome cascade, a biomarker that plays a crucial role in muscle function by eliminating sarcomere proteins. The UPS intensifies protein degradation processes, causing muscle fibres to lose proteins. Muscle-specific E3 ubiquitin ligases, including ‘atrogenes’, atrophy-related genes governing muscle component loss^9^ MuRF1 and MAFbx, become upregulated during atrophy, targeting structural proteins and contractile elements within muscle fibres^10^. Cellular signalling pathways, such as the Akt/mTOR pathway regulating protein synthesis, are also impacted, which additionally influence lysosomal pathways^11^. Atrogin-1/MAFbx genes also control MyoD degradation and eIF3f (essential for protein synthesis), and Muscle Ring Finger 1/ MuRF1, which regulate the half-life of various muscle structural proteins^12^. Studying muscle atrophy in space has provided valuable insights into muscle physiology and potential interventions. These mechanisms are pivotal for devising strategies to prevent or alleviate muscle atrophy through exercise, proper nutrition and targeted therapeutic interventions.

**Fig. 1:**
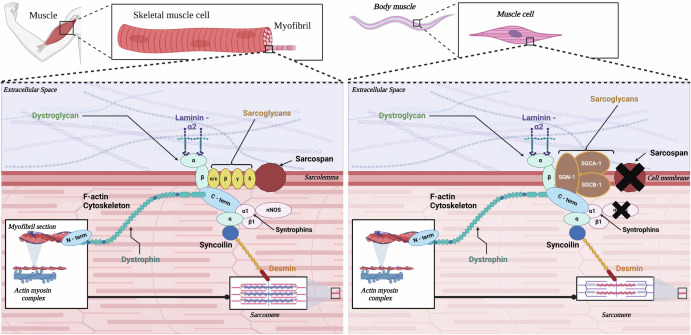
The dystrophin-associated glycoprotein complex in human (left) and *C. elegans* (right) muscle. Dystrophin binds to cytoskeletal F-actin and provides a mechanical connection between the actin cytoskeleton and the extracellular matrix via interacting with various DGC proteins. *Sarcoglycans***:** α-SG (LGMD2D), β-SG (LGMD2E), γ-SG (LGMD2C), δ-SG (LGMD2F), ε-SG (Myoclonus-dystonia), *Laminin***:** α2 (MDC1A), *Dystroglycan***:** α-DG, FCMD, MDC1C, *Desmin***:** related myopathies, *Dystrophin***:** Duchenne muscular dystrophy, Becker muscular dystrophy. Created with BioRender.com.

Molecular biomarkers (Table 1) are indispensable tools for tracking the intricacies of muscle atrophy, shedding light on its underlying mechanisms and monitoring its progression over time. Within the context of skeletal muscle tissue, a significant participant is the dystrophin-associated glycoprotein complex (DGC)^13^. Principally, the DGC functions to preserve the integrity of cell membranes, thereby reinforcing them against the strains and stresses incurred during muscle contraction and relaxation^14^. This stabilising function is indispensable for preserving the structural integrity of muscle fibres, thereby ensuring their capability to endure mechanical forces and sustain proper functionality over prolonged periods. In addition to its role in membrane stabilisation, the DGC operates as a sophisticated sensor system, proficient in detecting and transducing mechanical stress signals to the nucleus of the muscle cell^15^. In humans, the intricate organisation and functions of the DGC have been extensively studied, offering valuable insights into the pathophysiology of muscle-related disorders, including muscular dystrophies and muscle atrophy. Comparative studies with model organisms such as *C. elegans* provide additional layers of understanding and perspective. While humans and *C. elegans* are vastly different in terms of size and complexity, they share fundamental molecular pathways and mechanisms underlying muscle function and adaptation^16^. For instance, studies have revealed remarkable similarities between the DGC in humans and its homologues in *C. elegans*, despite the evolutionary distance between the two species^17^ (Fig. 1).

**Table 1:** Molecular biomarkers associated with muscle atrophy

Biomarker	Weight	Description
MuRF1 (Muscle RING Finger 1)	40 kDa^110^	An E3 ubiquitin ligase expressed in skeletal and cardiac muscle tissues in muscle remodelling. Upregulation participates in skeletal muscle atrophy; downregulation leads to cardiac hypertrophy.
Atrogin-1/MAFbx (Muscle Atrophy F-Box)	~42 kDa^111^	Muscle-specific F-box protein, when induced in catabolic states it is linked to muscle atrophy. Expression is detectable before muscle loss. Promotes muscle protein degradation by the ubiquitin-proteasome pathway.
Myostatin	~43 kDa^112^	Acts as myokine, negatively regulating Akt pathway, promoting protein synthesis, increasing ubiquitin-proteasome system activity to induce atrophy.
FoxO1	~70 kDa^113^	FOXOs induce the ubiquitin ligase Atrogin-1 and cause skeletal muscle atrophy.
FoxO3	~71 kDa^114^	
FoxO4	~65 kDa^115^	
Insulin-like growth factor-1 (IGF-1)	~8 kDa^116^	Muscle atrophy via the combined effects of altered protein synthesis, UPS activity, autophagy and muscle regeneration.
C-reactive protein (CRP)	~120 kDa^117^	CRP influences muscle cell size linked suppression of Muscle protein synthesis pathway.
Interleukin-6 (IL-6)	~21 kDa^118^	IL-6/gp130/JAK2/STAT3-pathway mediates sepsis-induced muscle atrophy.

Biomarker molecular weight and description of key biomarkers in disease state. Based on:^119^.

### Muscle degradation in astronauts

In microgravity, diminished muscle tension exacerbates spaceflight-induced muscular atrophy^18^, which may have multiple causes, such as muscular unloading and protein synthetic maintenance. However, the connection between muscle atrophy and protein degradation in astronauts during space missions was disproven in astronauts and later confirmed both in vitro using osteoblasts and in vivo using *C. elegans*^19^. This research has expanded understanding of the impact of microgravity on muscle physiology, providing critical insights into the intricate mechanisms that regulate muscle loss and the subsequent challenges encountered when navigating the demanding conditions of space travel. This link provided critical insights into the complex mechanisms that cause muscle mass decrease in space. Highlighting the importance of studying the relationship between muscle atrophy and protein breakdown to better understand metabolomic pathways in muscle degradation. A decrease in muscle protein synthesis (MPS) and/or an increase in muscle protein breakdown (MPB) can be a route cause of muscle loss in astronauts^20^. These processes are essential for the maintenance and regulation of muscle mass. In otherwise healthy muscle, muscle protein turnover is balanced, with MPS generally matching MPB, resulting in stable muscle mass. In the space environment, the equilibrium between muscle protein synthesis and breakdown is disrupted, resulting in a negative net balance, where breakdown exceeds synthesis. This imbalance leads to a loss of muscle nitrogen, which can occur due to an upsurge in breakdown or a reduction in synthesis^21^. In relation to MPS, FAK (Focal Adhesion Kinase) is a protein essential for mechanotransduction pathways, functioning at focal adhesions where cells connect to the extracellular matrix. During spaceflight, astronauts encounter microgravity conditions, disrupting the mechanical forces acting on cells and tissues^22^. This disturbance can impact FAK activation and other signalling molecules responsible for governing muscle growth, maintenance and repair, and impair anabolic signalling.

The ATP-dependent UPS plays a crucial role in regulating the degradation of myofibrillar proteins in response to muscle disuse, such as during prolonged periods of weightlessness in space^23^. This system is responsible for tagging proteins for degradation by the attachment of ubiquitin, marking them for recognition and subsequent breakdown by the proteasome - a cellular structure responsible for protein degradation^24^. During spaceflight, the lack of gravitational loading on muscles leads to decreased mechanical stress and disuse, triggering adaptive responses in muscle cells. One such response involves an upregulation of the UPS, resulting in an increased ubiquitination of proteins within the muscle fibres^25^. This increased ubiquitination targets myofibrillar proteins, which are the structural components of muscle fibres responsible for generating force and contraction^26^. This potentially contributes to muscle atrophy, or the wasting observed in astronauts during extended space missions.

### Muscle atrophy and biomarkers in space

It is known that astronauts suffer from muscle atrophy during spaceflight. This loss of muscle mass can be seen within in the muscle fibre showing a decrease between 2% and 19% in diameter and a 39% reduction in the width of myofibrils^27^. There is also a 79% loss of length in the Z-band, a structural component within a sarcomere, contributing to the organisation and function of muscle fibres, expressed within the myosin heavy chain (MHC)^28^. From previous strength training and muscle biopsy over a long duration mission (≥6-month period) with fast-twitch fibres show more significant atrophy. This affects astronauts’ ability to perform physical tasks during spaceflight and after returning to Earth. To mitigate muscle atrophy, astronauts participate in exercise programmes that include resistance training, aerobic exercise and neuromuscular training, which contributes to improved coordination, balance, strength and injury prevention^29^. Although mitigating muscle atrophy is not imperative for sustaining the health and performance of crew members during space missions, as indicated by NASA’s red list^30^, it could emerge as a concern with the advent of space commercialism and tourism. As private companies increasingly facilitate public space travel, the relevance of addressing muscle atrophy may become more pronounced, necessitating a closer examination of its potential impact on the wellbeing and capabilities of individuals venturing into space. Depending on factors like duration, physical activity levels and individual variability, the rapid onset of atrophy can occur within days or weeks of exposure.

**Fig. 2:**
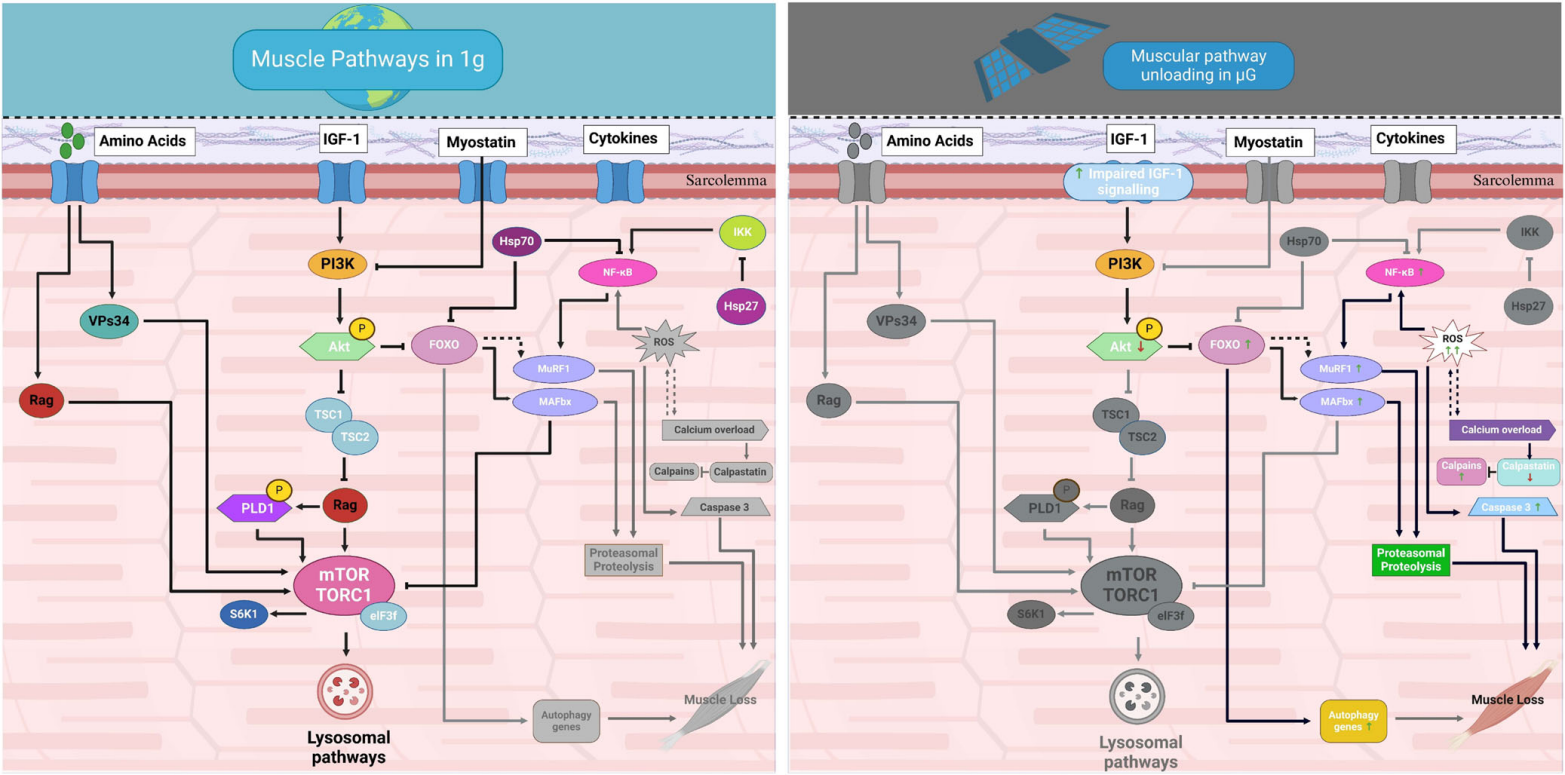
Changes contributing to reduced muscle strength in 1 G (left) and µG (right). Changes at a cellular level in response to muscle unloading in experienced in microgravity resulting in muscle atrophy in humans. IGF-1 insulin-like growth factor-1, PI3K phosphatidylinositol 3 kinase, Akt/PKB protein kinase B, regulated in DNA damage and development, ROS reactive oxygen species. The arrows and inhibition symbol show the association of molecules under loading condition, ↑ shows upregulation and ↓ downregulation of molecules under reduced gravity condition. Created with BioRender.com.

A 17-day spaceflight investigation using rats as a model organism found that during spaceflight, skeletal muscle mass loss leads to IGF signalling being impaired^31^. A bed rest study simulating muscular unloading recognised signalling mechanisms responsible of skeletal muscle atrophy following mechanical unloading. (Fig. 2)^32^. Studies on muscle atrophy and biomarkers in space have also utilised *C. elegans* as a model organism, subjecting the nematodes to spaceflight conditions aboard the International Space Station (ISS) or simulated microgravity environments^33^. Researchers have analysed alterations in gene expression, protein levels and physiological traits associated with muscle health and function in response to space conditions^34^. These investigations yield understanding of the molecular mechanisms driving muscle atrophy and aid in the identification of prospective biomarkers for evaluating muscle health during space missions.

Prolonged exposure to microgravity leads to changes in muscle fibre composition, a decrease in fast- twitch fibres and an increase in slow-twitch fibres^35^. Results of microgravity on the musculoskeletal system can vary based on factors such as gender, mission duration and results showing considerable variations between individuals. In a microgravity environment, force on muscle and bone is significantly reduced, leading to muscle atrophy, increased bone resorption, lower bone mineral density, and higher fracture risks^3^. Astronaut skeletal muscle undergoes physical changes during spaceflight, resulting in muscle exhaustion, lethargy, discomfort and lack of dexterity in activities due to the loss of muscle mass, which begins within the first 2 weeks of spaceflight and continues to degrade across longer missions (3–6 months)^36^. Muscle wasting, insomnia, pain and loss of dexterity all have a direct impact on physical performance. Astronauts rely on their physical ability to carry out their job, which varies from the mundane to the ability to respond to emergencies. These physiological issues highlight the need of understanding and resolving the impact of spaceflight on skeletal muscle to ensure astronauts’ wellbeing and performance throughout extended Lunar or Mars missions. However, these changes can reverse once subjects returned to 1 *g*^18^.

### Mitochondrial dynamics in microgravity

Numerous studies have revealed detailed molecular and cellular changes in response to microgravity exposure. DNA microarray research revealed considerable downregulation of major muscle components, cytoskeletal elements, metabolic genes and mitochondrial electron transport genes in microgravity settings, indicating a wide range of cellular responses. Despite this general trend, cardiac and skeletal muscles show tissue-specific differences in the regulation of nuclear-encoded mitochondrial proteins, indicating a more complex response to microgravity-induced stress^37^. Additionally, in simulated microgravity conditions, muscle cells struggle to maintain mitochondrial homoeostasis and begin to increase efforts at the expense of protein synthesis, highlighting mitochondria’s crucial role in cellular adaptation to altered gravitational pressures^37^.

While DNA microarray analysis indicates that major muscle components, cytoskeletal elements, metabolic genes and mitochondrial electron transport genes are largely downregulated in microgravity, cardiac and skeletal muscles exhibit tissue-specific variations in the regulation of nuclear-encoded mitochondrial proteins^38^. Under simulated microgravity conditions, cardiomyocytes strive to maintain mitochondrial homoeostasis at the expense of protein synthesis^39^. Furthermore, alterations in the cardiovascular system manifest in endothelial cells, contributing to the adaptation of the endothelium to gravitational unloading through mitophagy^40^. Metabolomic and proteomic analyses reveal changes in metabolic pathways, oxidative stress responses and disturbances in adenosine triphosphate (ATP) synthesis, indicating that microgravity may impair bone cell functions by compromising mitochondrial energy potential^41^.

**Fig. 3:**
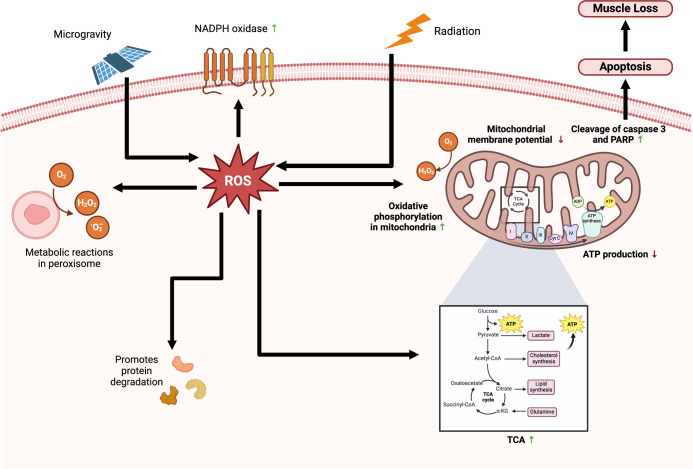
Reactive oxygen species (ROS) in microgravity. Microgravity environments like space cause oxidative stress, which influences mitochondrial processes such as glycolysis, tricarboxylic acid cycles, TCA, ROS levels and NADPH oxidase activity, leading to further protein degradation and apoptosis. ↑ indicates upregulation ↓ indicates downregulation. Created with BioRender.com.

The production of reactive oxygen species (ROS), a byproduct of mitochondrial ATP production, occurs during the process of electron transfer^42^. Microgravity conditions experienced in space lead to oxidative stress, influencing mitochondrial functions, including glycolysis, tricarboxylic acid cycles, ROS levels and NADPH oxidase activity^37^. These modifications result in the upregulation of processes such as glycolysis, while concurrently downregulating oxidative phosphorylation, ATP production and components of the mitochondrial respiratory chain^43^. Previous observations revealed changes in mitochondria-associated metabolites and modifications in nuclear DNA and mitochondrial DNA oxidative phosphorylation (OXPHOS) gene expression^44^. Imbalances in the assembly of OXPHOS complexes were hypothesised to trigger the mitochondrial unfolded protein response (UPRMT) and subsequently activate the integrated stress response^44^ Fig. 3.

## Harnessing *C. elegans* as a model organism for microgravity-induced muscle degradation in space research

### Modelling whole organism biology with *C. elegans*

*Caenorhabditis elegans* (*C. elegans*) is a microscopic nonparasitic nematode measuring ~1 mm in length. Its larvae hatch at around 0.25 mm and grow in size through various developmental stages^45^. From the 1940s^46^ to the present, *C. elegans* has appeared as a leading model organism for eukaryotic research. Though popularised in the 1970s by Sydney Brenner^47^. This prominence is due to its consistent cell count and easily mapped anatomy, which allow for the study of biological and biochemical interactions, metabolic processes and as well as modelling of ageing^48^. *C. elegans* is resistant to a wide range of environmental conditions, requires little space and is easily manipulable. Its adaptability extends its utility to a wide range of research areas^49^, biological and medical investigations, such as those related to reproduction, ageing, muscle atrophy, radiation effects and gene-protein interactions^50^. Recent research provides highly reproducible gene profiles across *C. elegans* are significantly associated with space-induced neuromuscular strength reduction, though further investigation is warranted into neuronal changes in calcium/acetylcholine signalling. These findings highlight the importance of using *C. elegans* as a model organism for multiple human diseases such as; Alzheimer’s, Parkinson’s and polyglutamine diseases, ultimately supporting medical efforts via providing an in vivo model to study before sending animals and humans further into outer space in the near future, such as extended missions to the moon and Mars, and how space related diseases can be investigated^51^.

As a model organism, *C. elegans* provides key benefits to research muscle atrophy and its underlying causes. Despite the evolutionary distance between humans and *C. elegans*, important biological processes and signalling pathways have been maintained. Muscle atrophy studies in *C. elegans* could present significant insights into biochemical mechanisms relevant to humans^52^. The details of the physiological and biological changes during muscle growth and degeneration are discussed below. Using nematodes as model organisms, particularly the extensively researched *C. elegans*, provides comprehensive details of their life cycle and development, allowing researchers to investigate the impact of altered gravitational conditions across various stages of nematode growth.

### Lifecycle and development

In comparison to the humans with an average lifespan of ~80 years, the life cycle of *C. elegans* displays notable differences in terms of duration and developmental stages. Within just 3 days at 20 °C, *C. elegans* undergoes a transition from an egg to an egg-laying adult. In contrast, the human life cycle spans multiple decades which is unfeasible when it comes to space travel research, making *C. elegans* an ideal model organism for space research. The life cycle of *C. elegans* is intricately divided into embryonic and postembryonic developmental phases, mirroring the structure of the human life cycle, which encompasses prenatal and postnatal developmental stages. However, the accelerated pace of *C. elegans* development is evident in its four larval stages (L1, L2, L3 and L4), each separated by a moulting phase before reaching adulthood^53^. Under adverse conditions, such as a lack of food or overcrowding, *C. elegans* also has the ability to enter a suspended L3 form known as the dauer larva, highlighting a unique survival strategy absent in the humans^54^. The dauer larva can endure adverse conditions for several months before resuming normal development when nutritional and environmental conditions improve^55^. This adaptability stands in contrast to the inability of humans to enter a suspended developmental state in response to adverse conditions.

In comparison to human reproduction in years, the reproductive characteristics of *C. elegans* demonstrate important differences. A self-fertilising hermaphrodite can produce around 300 fertilised eggs alone in a lifetime. If mated with a male, 1200–1400 fertilised eggs are produced. Male *C. elegans* can begin to mate for 6 days once adulthood is reached and can fertilise ~3000 eggs^56^. However, males are exceptionally rare, occurring at a rate of less than 0.2% for the standard lab strain N2^57^. Embryogenesis of *C. elegans* begins up to 350 min after insemination at 20 °C, cell divisions range from a single cell to around 550 stem cells^58^. This stage is further divided covering from zygote formation to embryonic founder cells, Hereafter, the majority of cell divisions and segmentation occur until organ development^59^. Throughout hermaphrodite maturation, 131 of the 1090 somatic cells undergo cellular senescence^60^. In adult *C. elegans* hermaphrodites, there are a total of 959 somatic nuclei, comprising 302 neurons and 95 muscle cells^61^. Initially, 81 muscle cells are present at hatching, with an additional 14 developing post-embryonically^62^. During larval development at the L1 stage, muscle cells are typically 2 sarcomeres wide and new sarcomeres are added, resulting in adult cells that may contain up to 10 sarcomeres in mid-body body wall muscle cells, with fewer sarcomeres in body wall muscle cells^63^. Concurrently, M-lines and dense bodies increase in size during post-embryonic development. In adult muscles, the contractile apparatus, ~1.5 μm thick, is aligned parallel to the hypodermis and the cuticle^64^. With a fixed number of cells, it is feasible to trace each cell from fertilisation to adulthood and construct a complete cell lineage, which has been reconstructed from electron micrographs^65^. Neuronal cells are predominantly clustered within several ganglia located in the head, ventral cord and tail regions. Although *C. elegans* primarily self-fertilise, male nematodes are found at a frequency of 0.2%^66^, possessing 81 additional neurons within their tails^67^. Approximately 80% of the genes of muscle attachment complexes in *C. elegans* are found in humans^68^. This positions *C. elegans* as an excellent model organism for space research, offering a versatile platform to study the effects of microgravity on specific anatomical features and physiological processes relevant to both nematodes and astronauts.

### *C. elegans* anatomy

Unlike most organisms *C. elegans* is optically translucent^69^ allowing for the observation of individual cells and subcellular features via differential interference contrast (DIC) optics. *C. elegans* contains an identifiable network of tissues and two concentric tubes, which the pseudocoelom a bodily cavity walled only partially by mesoderm-derived tissue, which houses multiple organs and allows nutrients and signals to circulate throughout the organism separates^70^. The exterior tube includes the cuticle, hypodermis, body wall muscle, nervous system and excretory system, whereas the interior tube includes the pharynx, intestine and reproductive system^71^. The epidermis is the outer layer of cells that encloses a pseudocoelomic fluid containing the primary organs^72^. The muscle bands that regulate mobility, alongside ventral and dorsal nerve cords which signal to the muscles, are found just beneath the epidermis. The gonad of *C. elegans* is a U-shaped region with an anterior gonad connecting to a posterior gonad at the midsection^73^. The digestive and excretory systems are located inside the neuromuscular area^72^.

#### *C. elegans* muscular anatomy

*C. elegans* has served as a valuable model for investigating muscle development, organisation and functionality. It possesses both striated and non-striated muscles. Non-striated muscles include various types such as 20 pharyngeal muscle cells, 2 stomatointestinal muscles, one anal depressor muscle, one anal sphincter muscle, 8 vulval muscles, 8 uterine muscles and 10 contractile gonadal sheath cells^74^. However, most studies have focused on the 95 striated body wall muscle cells, which are functionally analogous to human skeletal muscles. The fundamental structure, composition and role of the sarcomere, the basic functional unit of muscle, are highly conserved between nematodes and vertebrates.

**Fig. 4:**
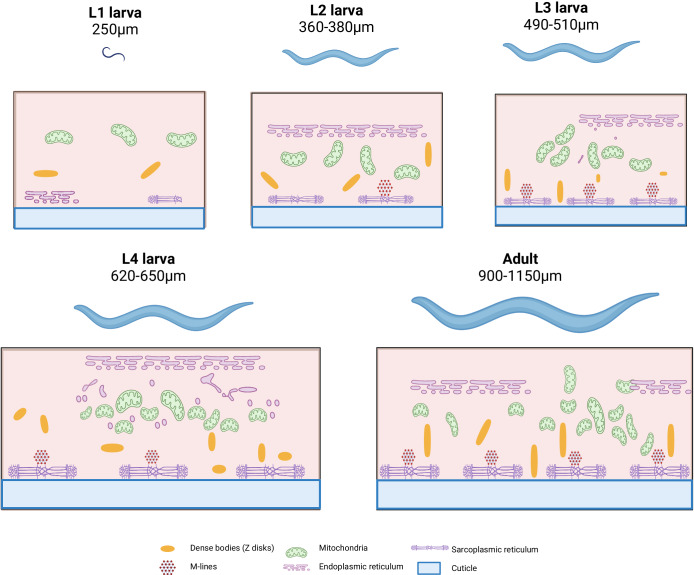
Life cycle of *C. elegans*, and relevant muscular changes with each stage. L1, newly hatched larvae show few mitochondria, dense bodies (Z-discs), ER, SR and no M-lines. L2, after 12 h larvae show an increase in mitochondria, dense bodies, ER, SR and first M-disk appears. L3, muscle begins to become more complex after an additional 8 h showing a further increase in mitochondria, dense bodies, ER, SR and M-discs. L4, after an additional 8 h mitochondria, dense bodies, ER, SR and M-discs increase again for the final larval stage. After 18 h the nematode has fully matured and muscle structure is completed with a final increase in mitochondria, dense bodies, ER, SR and M-discs. This artistic illustration is based on data presented in Gieseler K et. al.‘s *C. elegans* cross-sectional electron microscope images^75^. Created with BioRender.com.

Most body wall muscle cells in *C. elegans* develop during embryogenesis, with cellular organelles such as the nucleus, mitochondria, endoplasmic reticulum and ribosomes localised in the deeper regions of the muscle cell (Fig. 4)^75^. Notably, *C. elegans* muscles lack satellite cells, precluding muscle regeneration. In adult worms, body wall muscles are post-mitotic, mononucleated and do not fuse^75^. The 95 diamond-shaped body wall muscle cells in adult worms form a single layer and are arranged in four longitudinal bands of two rows each, termed quadrants, running from head to tail^76^. Each muscle cell contains multiple muscle fibres, typically 4–5 or more, with contractile fibres oriented in the anterior–posterior direction, exhibiting curvature corresponding to the body shape at the head and tail ends^77^.

In contrast, the male anatomy differs from that of the hermaphrodite, featuring a single-armed somatic gonad comprising 55 cells, as well as 41 specialised muscles, 79 additional neurons (including 87 sex-specific ones), 36 extra neuronal support cells (socket and sheath cells), 23 proctodeal cells and 16 hypodermal cells associated with mating structures, most of which are in the tail region^78^. Both sexes undergo four larval stages (L1-4), with sexual dimorphism becoming visually apparent during the L4 stage^79^.

### Research value of *C. elegans* for the study of microgravity’s biological impacts

The first instance of subjecting *C. elegans* to spaceflight was via a CNES (Centre National d’Etudes Spatiales) mission researching space medicine and biology^80,81^. These investigations were known as ICE-FIRST (International *Caenorhabditis elegans* Experiment - First - Aging [ICE-First Aging])^82,83^. This valuable data was reanalysed by Cahill et al. revealing new insights into molecular signatures of *C. elegans* in comparison with fast and slow twitch muscles during spaceflight^84^. This demonstrates that *C. elegans* is a valuable model organism for understanding human space missions and combating the adverse health effects of the space environment. Despite its simplicity as an organism, *C. elegans* shares numerous biological similarities with humans^85^. This encompasses genetic pathways, cellular processes and responses to environmental stressors, offering valuable insights into fundamental biological processes relevant to humans when studied in space allowing for precise manipulation of its genome, facilitating studies into specific genes and pathways involved in space adaptation and response to microgravity^84^. The organism’s transparent body permits clear visualisation of internal structures and physiological processes, simplifying the study of changes in muscle, nerve and other tissues under space conditions. These effects were measured through RNA sequencing^86^, microarrays^87^ and pathway analysis^86,88^. The reference strain for *C. elegans* wild-type is the Bristol N2 strain, most used when compared to other strains of nematodes subjected to spaceflight due to its well-characterised genome, genetic stability, ease of maintenance, short life cycle, availability of mutant strains and compatibility with various experimental techniques. Therefore, the results may be biased when drawing experimental conclusions^89^.

*C. elegans* serves as a valuable model organism or space research, supplying a unique opportunity to delve into muscle degradation studies under microgravity conditions, providing insights that bridge the gap between fundamental biological processes and the challenges faced extraterrestrial environments.

#### *C. elegans* muscle degradation studies

Both humans and *C. elegans* share fundamental mechanisms for muscle atrophy and protein degradation, particularly through the involvement of common cellular processes and signalling pathways such as the insulin-like growth factor (IGF-1)/Akt pathway known to influence muscle protein synthesis and degradation. In the ICE-FIRST study, *C. elegans* underwent changes in genes and proteins related to worm musculature which was measured via microarray analysis and 2D gel electrophoresis and MALDI-TOF MS^90,91^. Microgravity has the potential to influence the synthesis of myosin transcription factors and genes, leading to atrophy in parietal muscle^92^. A decrease in muscular contractility results in significant motility abnormalities. The spaceflight response in *C. elegans* is triggered by reduced myosin heavy chain expression in both the body wall and the pharyngeal muscle, facilitating changes in motility, feeding and pseudocoelomic circulation^93^. Microgravity can also lead to an increase in the expression of genes associated with early embryogenesis^94^. Conversely, the expression of genes related to locomotion decreased under microgravity conditions^95^. CeMyoD and HLH-1, crucial for muscle activity and morphogenesis, exhibit reduced expression in space-flown *C. elegans*^95^. This decrease correlates with diminished levels of *myo-3* and *unc-54* genes, encoding MHC *a* and paramyosin, in body wall muscles. moreover, pharyngeal myogenic transcription factors *peb-1, ceh-22* and *pha-4* also show decreased expression^96^. In *C. elegans* adapting to space aboard the ISS, MHC isoforms A and B in body wall muscles and isoforms C and D in pharyngeal muscles, are downregulated compared to ground controls. Likewise, the expression of transcription factors regulating myosin heavy chain isoforms decreases. Troponin and tropomyosin genes are also downregulated. Previous space-flown *C. elegans* display decreased expression of the myogenic transcription factor HLH-1 and increased expression of *skr-6* and *skr-18*, ubiquitin ligase enzymes promoting protein degradation. additionally, decreased expression is observed in *myo-1, myo-2*, and *unc-54* genes encoding MHC *d, c* and *b*, as well as *unc-15* genes and genes encoding troponins and tropomyosin’s *(tnc-2, tnt-2, tnt-3, tnt-4 and lev-11)*. These alterations in gene expression lead to muscle atrophy in response to spaceflight^97^. (Table 2).

**Table 2:** *C. elegans* CeMyoD expression is reduced during spaceflight

Gene	Downregulation	Encoding
*myo-3*	73%	MHC-A and Paramyosin
*unc-54*	22%^a^	
*peb-1*	16.7%	Pharyngeal myogenic transcription factors ^α^
*ceh-22*	70.5%	
*pha-4*	51.8%	
*myo-1*	10%^b^	MHC-D, C and B
*myo-2*	49%	
*unc-54*	10%	
*unc-15*	87.8%	Troponins and Tropomyosin’s
		*tnc-2*
		*tnt-2*
		*tnt-3*
		*tnt-4*
		*lev-11*

*PEB-1* zinc finger-containing protein, *CEH-22* NK-2 class homeoprotein, *MHC-A/D/C/B* major histocompatibility complex, *TNC-2* Tenascin C-2, *TNT-2* Troponin-2, *TNT-3* Troponin-3, *TNT-4* Troponin-4, *LEV-11* Tropomyosin isoforms a/b/d/f. *Myo1* Myosin-1, *Myo2* Myosin-2, *Myo3* Myosin-3, *UNC-54* UNCoordinated locomotion encoding major body wall muscle, *MHC* Myosin heavy chain.

^a^Body and ^b^pharyngeal muscle specific^120,121^.

The findings of *C. elegans* muscle degradation investigations conducted in microgravity experimental contribution of nematode physiology, but also provide vital insights applicable to broader study investigating the consequences of changing gravitational conditions.

## Unveiling microgravity’s impact with insights from simulated environments and model organisms

The effects of the space environment can be dangerous. Therefore, space environment simulations/analogues are the best chance at monitoring the certain different hazards such as: microgravity, cosmic radiation, atmospheres of other celestial bodies. Identifying the effects of microgravity is crucial for space exploration, as it allows scientists to develop countermeasures and strategies to mitigate these effects.

The foundation for detecting the physiological effects of weightlessness is laid by general microgravity experimentation and the insights gained from such studies often inform and supplement research conducted under simulated microgravity conditions, creating a comprehensive framework for investigating the diverse impacts of altered gravitational environments.

### Technical simulated microgravity

**Fig. 5:**
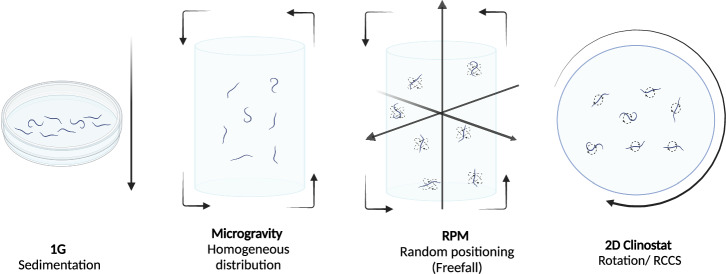
Microgravity simulators. Representation of *C. elegans* nematodes subjected to the rotational movement of different microgravity simulators when cultured in liquid media. Created with BioRender.com.

There are four main laboratory devices for recreating some of the environmental properties of microgravity on Earth to examine its impact, including, diamagnetic levitation, rotating wall vessels, two-dimensional clinostats (2-D), three-dimensional (3-D) clinostats also known as random positioning machines (RPMs) (Fig. 5)^98^.

Constant reorientation of the gravity vector, using devices like a 2D or 3D clinostat, is a method to average out the effects of gravity when the system’s evolution or growth is slower than the rotation rate. This technique is commonly used to study the impact of gravity on plant growth by growing plants sideways on a 2D clinostat. Similarly, a 3D clinostat or RPM can average out the gravitational vector, which is particularly useful for studying objects with distinct orientations^99^.

Weightlessness occurs when an object accelerates at the same rate as the local acceleration due to gravity. For example, an astronaut standing on the Earth’s surface experiences an acceleration due to gravity of 9.8 m/s²^100^. In contrast, an astronaut on the ISS accelerates towards the Earth at 8.9 m/s², matching the gravitational acceleration in Low Earth Orbit^101^. At the centre of an RPM, an object is not accelerating, though it still experiences a gravitational acceleration of 9.8 m/s²^102^.

Samples in liquid within an RCCS or RPM continuously fall towards the Earth, causing them to sediment, while the vessel rotates, creating micro-orbits. This movement results in the sample travelling through the fluid at a constant velocity, leading to a low shear environment. When these samples and their vessels are placed on the ISS, both accelerate together, preventing the sample from moving through the fluid^68^.

### Biological simulated microgravity effects

Other techniques such as hindlimb unloading, a technique that involves unweighting the hindlimbs of rodents, effectively simulates aspects of microgravity. This model is especially useful for studying muscle atrophy and has been widely used to investigate the effects of altered loading on skeletal muscle in the context of space-related research^103^. Similarly, using rotating wall vessel bioreactors as in vitro simulations results in a low-shear culture environment like that of microgravity. Such systems are useful for studying muscle cells in vitro, allowing researchers to better understand the effects of different mechanical loading on muscle tissue. In vitro simulations, such as 3D cell culture models, also provide a way to closely mimic the in vivo microenvironment. By replicating three-dimensional cell cultures, these models provide a platform for investigating the mechanisms of muscle atrophy at the cellular level, including changes in muscle cell structure and function^104^.

*C. elegans* has been vital in space experiments, allowing researchers to study muscle changes and dissect metabolic pathways under the extreme conditions of space^87^ playing a pivotal role in space experiments, facilitating the study of muscle changes and the dissection of metabolic pathways under the unique conditions of space. Additionally, in vitro simulations such as 3D cell culture models offer a means to closely mimic the in vivo microenvironment. These models, by replicating three-dimensional cell cultures, provide a platform to delve into the mechanisms of muscle atrophy at the cellular level, including alterations in muscle cell structure and function.

Model organisms, such as *C. elegans* and mice, are subjected to these simulated microgravity environments to study changes in muscle structure and function^105^. Muscle atrophy mechanisms can also be studied at the cellular level by cultivating muscle cells in conditions that mimic reduced load, such as using special scaffolds or growth media. Previous research has demonstrated that exposing *C. elegans* to 2D clinostat simulated microgravity can cause oxidative stress and response pathways within worms^106^, which may negatively influence the result outcomes. *C. elegans* can be effectively cultured within simulated microgravity method. Culture of *C. elegans* provides a significant benefit in determining if microgravity environments are achieved in a 2D clinostat filled with medium, causing the nematode to ‘free-fall’ within the fluid. According to formerly reported microgravity gene expression research, a velocity of 20 rpm reliably promotes *E. coli* gene expression^107^. Adult nematodes subjected to artificial microgravity exhibited reduced brood sizes than 1 *g* experiencing adult nematodes. In static conditions, wild-type *C. elegans* N2 nematodes can have a brood count of roughly 217 larvae, whereas wild-type simulated worms have a decrease in brood population^108^. This was true regardless of dietary content or condition, implying that something other than nutritional insufficiency may be to blame. The reproductive organs of control and simulated microgravity nematodes can be visualised to acquire a clearer insight of the diminished reproductive ability in microgravity. These findings appear comparable to those found in adult reproductive diapause (ARD), a condition wherein adult hermaphrodites defer reproduction and live longer in relation to malnutrition^109^.

Therefore, the use of rotating wall vessel bioreactors as in vitro simulations creates a low-shear culture environment akin to aspects of microgravity. Such systems prove valuable for studying muscle cells in vitro, allowing for an understanding of the impact of altered mechanical loading on muscle tissue. Simulated microgravity experimentation is a valuable tool for unravelling the complex consequences of altered gravitational conditions, providing valuable insights that contribute to knowledge of physiological responses and paving the way for the development of targeted interventions in a variety of scientific fields.

### Concluding remarks

This review emphasises the critical importance of using model organisms to investigate the complex challenges posed by long-duration spaceflight. Earlier studies have shed light on the impact of microgravity, however, the possibility of long-term chronic health deficits in astronauts remains uncertain. In this context, the use of nematodes, as a valuable tool for assessing muscle atrophy in simulated microgravity conditions, emerges as an important avenue for unravelling the nuanced physiological effects of space travel. The importance of this field of research is highlighted by its direct relevance to determining astronaut health status during extended space missions and anticipating the wellbeing of future space travellers in the burgeoning era of space commercialisation. Recognising the amount of knowledge that can be gained by scrutinising previous approaches to studying muscle atrophy and overall bodily changes during long-term space travel, for which *C. elegans* nematodes is an ideal system to study. By delving into the complexities of muscle atrophy methods and suggest advancements in the context of *C. elegans* research, it is anticipated that this will pave the way towards refinement of research methodologies associated with extended periods of spaceflight.
